# Study on Material Removal Mechanism of Non-Resonant Vibration-Assisted Scratching High-Volume Fraction SiCp/Al

**DOI:** 10.3390/mi16040360

**Published:** 2025-03-21

**Authors:** Yuan Xi, Yan Gu, Jieqiong Lin, Zisu Xu, Zhiduo Fan, Tianyu Gao, Xiaoming Zhang, Yuanshuo Liu

**Affiliations:** 1 Jilin Provincial Key Laboratory of Micro-Nano and Ultra-Precision Manufacturing, School of Mechatronic Engineering, Changchun University of Technology, Yan’an Ave 2055, Changchun 130012, China; 13944475509@163.com (Y.X.); linjieqiong@ccut.edu.cn (J.L.); 15568607008@163.com (Z.X.); 15636410515@163.com (Z.F.); 15981025677@163.com (T.G.); zxm20011019@163.com (X.Z.); thorigen@163.com (Y.L.); 2 Jilin Province Key Laboratory of International Science and Technology Cooperation for High Performance Manufacturing and Testing, School of Mechatronic Engineering, Changchun University of Technology, Yan’an Ave 2055, Changchun 130012, China

**Keywords:** non-resonant vibration, material removal mechanism, SiCp/Al, molecular dynamics

## Abstract

Aluminum-based silicon carbide (SiCp/Al) is a hard-to-process material. SiC particles are randomly distributed and have a unique structure, thus posing challenges during processing. These distinctions considerably affect the overall quality of machining. As the volume fraction increases, the machinability continues to decline. Understanding the removal mechanism of SiCp/Al composites is essential for improving their machined surface quality. This study explores the influence of vibration frequency on the removal mechanism and plastic deformation in high-volume fraction SiCp/Al composites using non-resonant vibration-assisted scratching (NVAS) experiments combined with molecular dynamics (MD) simulations. The experimental results show that compared with conventional scraping (CS), increasing the vibration frequency in the NVAS process significantly expands the plastic removal area and reduces the scraping force. The simulation results indicate that as vibration frequency rises, the smoothness of the scratched groove improves, leading to a more uniform distribution of dislocations and a significant reduction in dislocation loops and HCP structures, promoting plastic deformation of the material. The simulation explains and clarifies the occurrence of plastic deformation observed during the scratching experiments. This study can provide a potential understanding of non-resonant vibration-assisted high-volume SiCp/Al composites machining.

## 1. Introduction

SiCp/Al materials are widely utilized in the aerospace, military, and electronic packaging fields owing to their unique features, which encompass high specific strength, heat resistance, and corrosion resistance [1,2]. The high-volume fraction SiCp/Al composites, with more than 50% SiC particles, are being explored as potential alternatives to traditional materials like microcrystalline glass, quartz glass, and silicon carbide for space mirrors [3]. SiCp/Al is a commonly encountered hard-to-machine material due to the considerable compositional variations between SiC particles and the Al matrix, resulting in various material removal mechanisms for the Al matrix and SiC particles. During the machining process, the aluminum matrix undergoes plastic deformation, while silicon carbide particles exhibit phenomena such as crushing, cracking, and debonding [4]. The random distribution of SiC particles in the Al matrix results in significant complexity in the removal mechanism of SiCp/Al composites. The scratch experiment provides an effective method to reveal the scratch removal mechanism in SiCp/Al composites by analyzing the scratch morphology and characteristics. This method has been adopted for hard-to-process materials such as SiC ceramics, CFRP, and C/SiC [5,6,7]. Scratch experiments can help adjust vibration parameters and depth of cut, reduce surface damage, and improve machining efficiency. The research results can be used to optimize the machining process for non-resonant vibration-assisted grinding, increase the material removal rate and improve the surface quality. This study can provide guidance for non-resonant vibration-assisted machining of difficult-to-machine composite materials.

In the CS process, Gu [8] et al. analyzed the material removal behavior of SiCp/Al by varying load CS with scratch loads in the range of 1 mN–400 mN. The findings indicated that plastic removal is the predominant method of removal during scratching and that increasing the normal force increases the scratching depth. The findings indicated that the material removal behavior of SiCp/Al composites is significantly different from that of SiC ceramics at macroscopic and microscopic scales, and the normal force has an important effect on the material removal behavior [9]. Simultaneously, studies demonstrate that the implementation of ultrasonic vibrations significantly improves material removal efficiency [10].

Ultrasonic vibration-assisted machining (UVAM) is an advanced technology with benefits such as decreased machining forces and enhanced surface quality [11]. Zheng [12] et al. analyzed the deformation and removal mechanisms of SiCp/Al materials by CS and ultrasound-assisted scratching (UVAS). The findings indicated that UVAS had both a stronger scratching force and a greater friction coefficient than CS. UVAS raised material removal effectiveness, and the damage of scratched surfaces was lower than that of CS in the UVAS process. Feng [13] et al. showed that ultrasonic vibration increased the cracks in SiC particles and promoted SiC removal by contrasting the deformation characteristics of SiCp/Al under rotational ultrasonic scratching (RUVAS) with those under CS. Xiang [14] et al. investigated the scratching profiles of SiCp/Al under UVAS and CS. The depths and heights of the scratching profiles under UVAS were greater than those under CS. The bottom of the scratching groove is smoother under UVAS. Currently, in UVAS, high frequencies dominate the ultrasonic vibrations commonly applied, and the applied ultrasonic vibration frequency cannot be changed. Current research lacks systematic investigations into the application of low-frequency vibrations and their frequency variations in the scratching process. Non-resonant vibration systems can vary the frequency and amplitude of vibration and are driven by PZT (piezoceramic), which allows precise transfer of motion and reduction in parasitic motion [15]. Prasad Prabhu [16] et al. developed a non-resonant vibration system, and the findings demonstrated that the increased frequency reduces the surface roughness.

The studies mentioned above indicate that vibration positively influences material removal. To elucidate the material removal mechanism influenced by vibration, it is essential to investigate its microscopic effects on materials. With advancements in computational technology, molecular dynamics (MD) simulations of nano-scratching offer a robust approach to analyze vibration-induced plastic deformation mechanisms. Huang [17] et al. explored the implications of varying vibration frequencies on the deformation mechanism of single-crystal iron. The findings indicated that introducing vibration decreased the thickness of subsurface damage and dislocation line length, and the increase in vibration frequency was beneficial for enhancing plasticity. Zheng [18] et al. explored the impact of varying distinct vibration settings on the microplastic deformation behavior of single-crystal copper. The findings indicated that high-frequency vibration reduced the scratching force and promoted plastic deformation, and two-dimensional vibration was conducive to achieving greater surface quality. Yang [19] et al. examined the plastic deformation behavior of copper under different vibration settings. The findings indicated that vibration increases the material’s plastic deformation ability and reduces the average force. Ban [20] et al. examined the impact of ultrasonic vibration on the removal mechanism of 4H-SiC material. The findings indicated that lower frequency and amplitude can improve surface quality. Huang [21] et al. examined the impact of different vibration parameters on the deformation mechanism of nanograin iron alloy. The findings indicated that high stress promoted the value-added dislocations, the formation of more deformation twins suppressed the dislocation activity under the strain rate effect, and the transverse dislocations and deformation twins jointly affected the plastic deformation. The studies mentioned above indicate that MD simulations are a valuable tool for investigating the mechanisms behind vibration effects. However, there is a scarcity of research focused on how vibration influences the plastic deformation mechanisms of SiCp/Al, which restricts their practical applications.

To address these research gaps, non-resonant vibration-assisted scratching experiments were performed to reveal the mechanism of material removal for SiCp/Al subjected to different vibration frequencies, in addition to non-resonant vibration-assisted scratching MD simulations, which revealed the plastic deformation mechanism of SiCp/Al under the action of different vibration frequencies and the MD simulations enhanced the understanding of the plastic deformation phenomenon at the atomic level during the scratching experiments.

The organization of the paper is summarized: Section 2 details the experimental setup and the MD simulation model, and Section 3.1 examines the surface morphology and scratching force of CS and NVAS. Section 3.2 examines how various vibration frequencies influence the microstructure of SiCp/Al composites through MD simulations, and Section 4 concludes the conclusions.

## 2. Materials and Methods

Vibration-assisted scratching experiments are needed to clarify the material removal mechanism of SiCp/Al. In order to further understand the mechanism of plastic deformation of SiCp/Al by observing how the interface between SiC particles and Al matrix interacts and how the particles affect the plastic deformation of the matrix under external forces, MD simulations are needed to be performed.

### 2.1. Setup of the Scratching Experiment

The samples used in this study were SiCp/Al (SiC particle volume fraction was 60%), and the dimensions of the samples were 1 cm × 1 cm × 1 cm; Xi’an Kurt New Materials Co. Ltd. (Xi’an, China). provided, and the manufacturing method was powder metallurgy. To ensure that the initial surface of the samples is not damaged so as not to affect the results of the subsequent scratching experiments, samples were meticulously ground and polished by means of a grinding and polishing machine (AutoPol GP-1A, Suzhou, China), and the surface roughness of the samples was measured by means of a white light interferometer (Zygo, NewView 8000, Middlefield, CT, USA) before performing the scratching experiments. The average roughness of the samples reached 70 nanometers. Then, the CS and NVAS trials were carried out.

**Figure 1:**
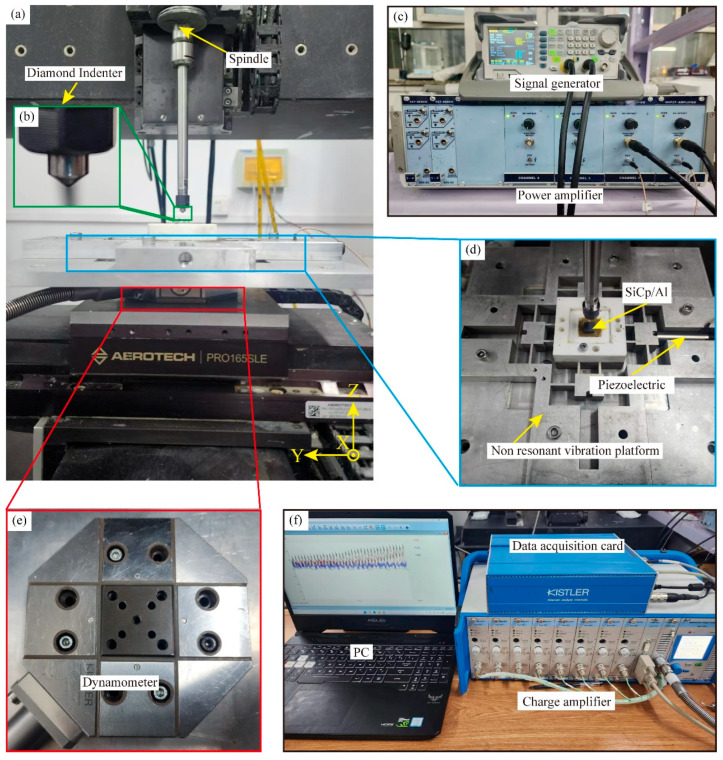
Experimental setup of NVAS. (**a**) Physical drawing of the machine. (**b**) Enlarged view of the diamond conical indenter. (**c**) Physical view of the signal generator and power amplifier. (**d**) Physical drawings of the non-resonant vibration stage, PZT, and SiCp/Al samples. (**e**) Physical drawing of the dynamometer. (**f**) Diagram of the data acquisition card, charge amplifier, and computer.

Figure 1 depicts the scratched experimental setup, with CS and NVAS studies carried out using an in-house machine tool, as embodied in Figure 1a. Figure 1b shows a diamond conical indenter installed on the machine tool’s spindle, which is an enlarged schematic diagram of the indenter (the indenter tip radius measures 120°). As embodied in Figure 1c,d, the non-resonant vibration stage, signal generator, power amplifier, and PZT form the vibration generation system. The signal generator generates a vibration signal. A power amplifier strengthens this signal and then sends it to the PZT, which activates the non-resonant vibration platform and allows the sample to experience non-resonant vibrations. The first-order intrinsic frequency of the non-resonant vibration platform is 1800 Hz, and the maximum displacement is 40 μm. As embodied in Figure 1e,f, the scratching force test system includes a computer, dynamometer, charge amplifier, and data acquisition card. The dynamometer is positioned beneath the non-resonant vibration platform. The host computer configures its sampling frequency to 1 K. The scratch force data generated during the scratch measurement process is collected in real time by the force sensor and displayed on the host computer.

To obtain accurate experimental results, three experiments were carried out for each set of experimental parameters. To maintain the stability of the scratching process, the scratching speed was selected to be 100 μm/s. For easy observation, the scratch time was chosen as 30 s. Due to the high degree of random distribution of SiC particles in high-volume fraction SiCp/Al in the Al matrix, the use of scratching experiments with a variable scratching depth may affect the analysis of the material removal mechanism, and a constant scratching depth was set in CS experiments, a large span of scratching depth parameters was selected, and the constant three scratching depth parameters are commonly used in processing experiments [22,23,24]. The specific parameters chosen in the experiment are embodied in Table 1. No. 1–3 recorded the experimental parameters of CS three times, and No. 4–6 recorded the experimental parameters of NVAS three times.

### 2.2. Modeling Details for MD Simulation

**Figure 2:**
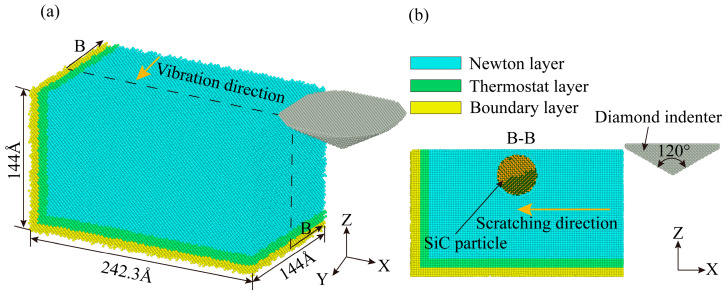
Non-resonant vibration-assisted scratching MD model.

**Figure 3:**
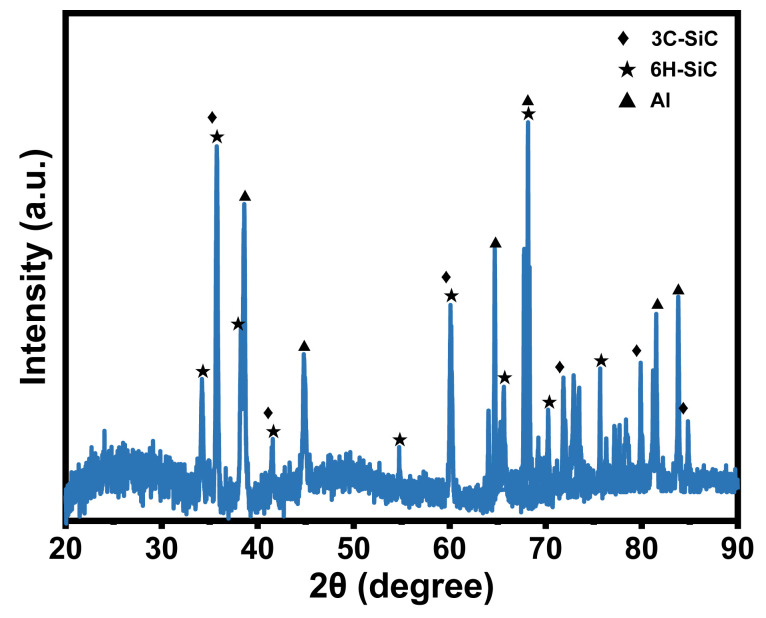
XRD test.

To analyze the plastic deformation mechanism of non-resonant vibration-scratched SiCp/Al material at the microscopic scale, the MD model of non-resonant vibration-assisted scratching of SiCp/Al was established. As embodied in Figure 2a, the MD model consists of a diamond indenter and a SiCp/Al sample, and the indenter makes a straight line scratching across the *x*-axis negative direction on the sample during the CS process. As shown in Figure 3, the XRD test on the samples shows that the crystal structures of SiC in SiCp/Al are 3C-SiC and 6H-SiC, with the 3C-SiC for study in MD simulation. During the NVAS process, the vibration direction is 90° from the scratch direction. The specific parameters used are embodied in Table 2. Higher speeds have a minor effect on MD simulations. In order to observe the diamond indenter in a short period of time, the speed of the indenter was set to be much greater than that of the scratch experiment. Similarly, the frequency was chosen to be greater than that of the scratching experimental procedure in order to achieve a significant vibration effect during this time [25,26,27], and the dimensions of the SiCp/Al sample are 242.3 Å × 144 Å × 144 Å. The simulated SiCp/Al sample can be categorized into three distinct layers: the Newtonian, thermostatic, and boundary layers. The motion of atoms in the thermostatic and Newtonian layers adheres to classical Newtonian principles [28]. The atoms in the boundary layer are immobilized to keep the sample in the proper position [29], and the thicknesses of the boundary and thermostatic layer are 10 Å. As embodied in Figure 2b, in MD simulations, the particles are usually considered spheres uniformly distributed in the Al matrix. In order to simplify the simulation, SiC was placed in the center of the Al matrix [30], and the SiC particles were set as spheres with a radius of 30 Å for the SiC particles. A diamond indenter with a 0.357 nm lattice is designated as a rigid body because it is much harder than the sample material. The angle of the tip of the indenter is set to be 120°, the same as that for the scratch experiment. Boundary conditions were applied periodically across the X, Y, and Z axes.

Using the NPT ensemble, a 30 ps relaxation was performed to stabilize the model at an initial temperature of 300 K. The time step is configured to 1 fs. The interaction (Si−Al, $C_{\mathrm{sic}}-Al$) between the Al and SiC atoms in the SiCp/Al sample and the interaction ($Si-C_{diamond}$, $C_{sic}-C_{diamond}$, $Al-C_{diamond}$) between the C atoms in the diamond indenter and all the atoms in the SiCp/Al sample are similarly expressed in the lj/cut potential, with the detailed parameters shown in Table 3, ε denotes the depth of the attractive well, σ denotes interparticle distance and $r_{c}$ denotes cut-off radius. The Vashishta potential function represents the interactions between SiC atoms [31]. The embedded atom method (EAM) potential depicts interactions among Al atoms [32].

The diamond indenter moves along the SiCp/Al sample with a velocity of v. The indenter, which is subjected to the action of a non-resonant vibrating platform, generates a sinusoidal motion in the Y-direction. The trajectory could be expressed as follows:

(1) $$\left\{\begin{array}{l} X=-vt\\ Y=A\sin(2\pi t/T) \end{array}\right.$$

where *v* represents scratching velocity, *A* denotes amplitude, *T* denotes vibration period, and *t* denotes time.

MD simulations simulated the scratching behavior of a diamond indenter using LAMMPS (2Aug2023) [33]. The diamond indenter and SiCp/Al sample models were constructed using the Atomsk (0.13.1) [34] program. The simulation results were visualized and analyzed by OVITO (2.9) [35].

## 3. Results and Discussion

The surface morphology and scratching force during CS and NVAS are analyzed by SEM and dynamometer in Section 3.1, and the cross-section of the scratched groove, hydrostatic stress distribution, dislocations, phase transition, dislocation density, and scratching force during CS and NVAS are analyzed by MD simulation in Section 3.2.

### 3.1. Implication of Vibration Frequency on Scratching Outcomes

Surface morphology reflects the microscopic changes in the surface of a material after it has been subjected to a localized external force. By observing the surface morphology, it can reveal the deformation and damage process of the material. The analysis of scratch forces is an important aid in understanding the plastic deformation as well as the damage behavior of materials.

#### 3.1.1. Surface Morphology Analysis

**Figure 4:**
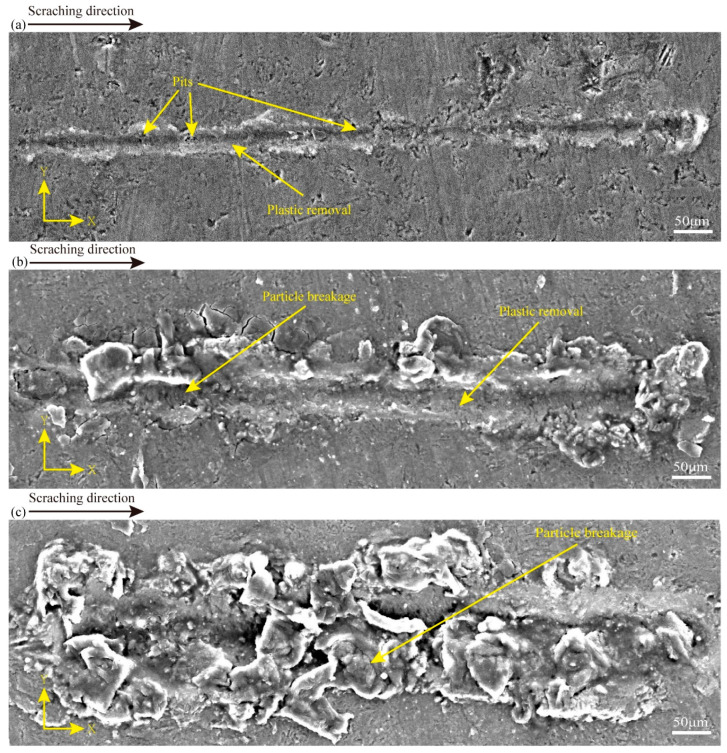
SEM images used to compare the surface topography of CS at varied scratching depths: (**a**) 0.2 μm, (**b**) 3 μm, (**c**) 6 μm.

To obtain insights into the material removal process of CS, the surface characteristics of the CS groove are embodied in Figure 4, and the processing parameters in Figure 4a–c are in line with experiments 1–3 in Table 1. Figure 4a shows that at a scratching depth of 0.2 μm, there are some pits in the scratching process. Also, there are more areas of plastic deformation throughout the scratching at a scratching depth of 3 μm. There are some particle crushing and plastic deformation. At a scratching depth of 6 μm, there are many particles crushed during the scratching process. It can be seen that there is a certain degree of surface damage in SiCp/Al at different scratching depths, and there is a different degree of plastic removal at 0.2 μm and 3 μm scratching depths, which can also prove that the variable scratching depths may affect the analysis in relation to how the material is removed (in this study, SiCp/Al removal occurs in a plastic removal mode when the SiC particle-reinforced phase and the Al matrix phase undergo simultaneous plastic deformation during the material removal process). In addition, many experimental data comparisons were carried out, and it was finally obtained that less surface damage was produced at a scratching depth of 0.2 μm. To examine the implications of varying vibration frequencies on the removal mechanism of SiCp/Al materials, a scratching depth of 0.2 μm was used in the subsequent NVAS experiments.

**Figure 5:**
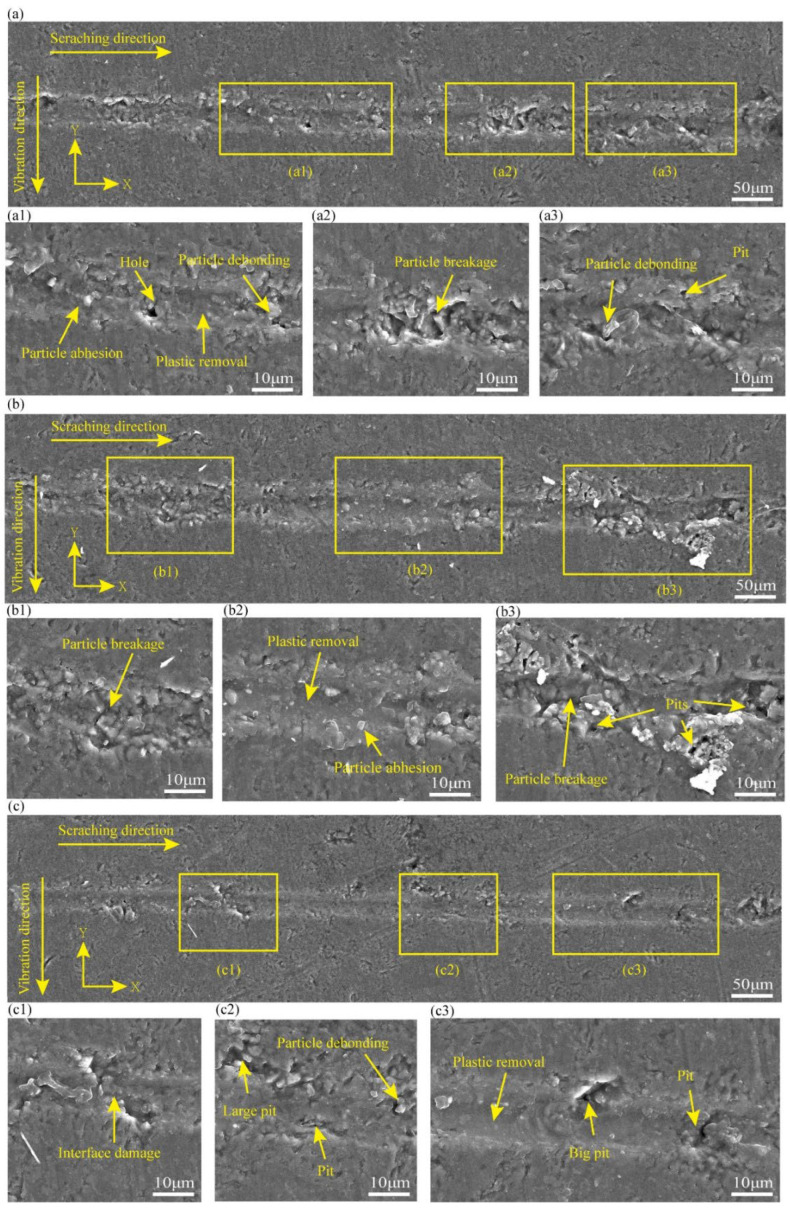
SEM images used to compare the surface topography of NVAS at varied vibration frequencies: (**a**) 30 Hz, (**b**) 60 Hz, (**c**) 120 Hz.

To characterize the material removal mechanisms of NVAS, the surface characteristics of the NVAS groove are embodied in Figure 5. The experimental parameters in Figure 5a–c are associated with experiments 4–6 in Table 2. As embodied in Figure 5(a1–a3), under the vibration frequency of 30 Hz, there are pits and fewer areas of plastic deformation in the scratching groove in the NVAS process. As embodied in Figure 5(b1–b3), under the vibration frequency of 60 Hz, the plastic deformation region is enlarged, particle crushing occurs, and small pits are present in the scratching process. As embodied in Figure 5(c1–c3), under the vibration frequency of 120 Hz, the removal behavior of NVAS is dominated by plastic removal, the surface of the scratched groove is smoother, there is interfacial damage, and the scratching process is accompanied by fewer pits and no particle crushing.

To summarize NVAS compared to CS1, which summarized that vibration changes the removal behavior of SiCp/Al. With the vibration frequencies of 30 Hz and 60 Hz, vibration improves the surface damage. It deteriorates the surface quality, and under the vibration frequency of 120 Hz, the region of plastic removal is improved. The phenomena of surface damage, such as pits and crushing, are significantly reduced, and the phenomenon of particle adhesion is less. This may be due to the applied scratching direction, the striking force generated by vibration acts on the side of the scratching, and at lower frequencies, the vibration cycle is longer, which may produce a localized stress concentration, resulting in brittle damage and improved surface damage. The enhanced vibration frequency extends the dynamic contact length of the sample with the indenter, leading to a higher percentage of plasticity removal [36].

#### 3.1.2. Scratching Force Analysis in CS and NVAS Processes

**Figure 6:**
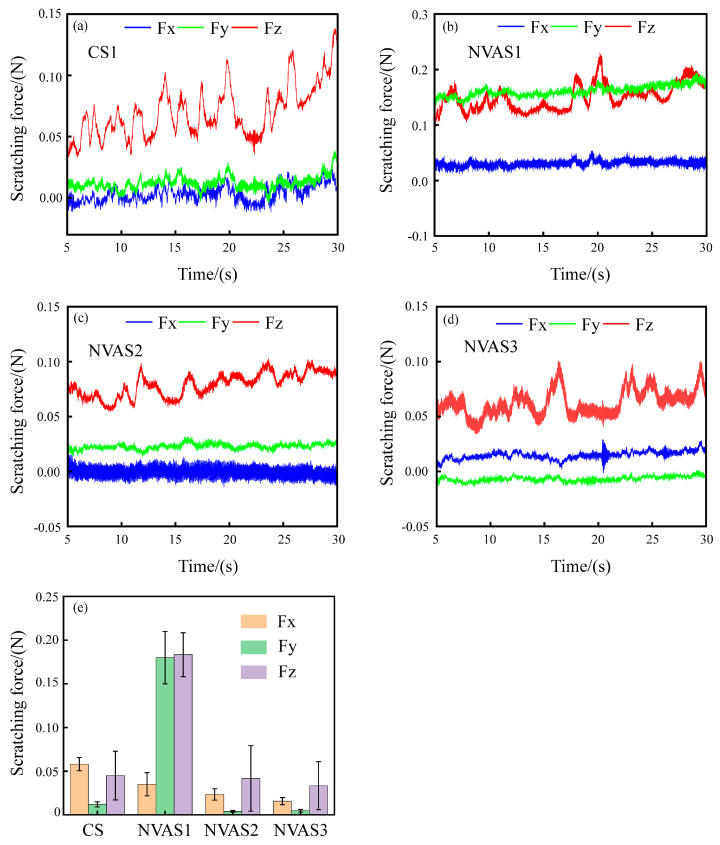
Fluctuation of scratching force during CS and NVAS, Fx tangential scratching force, Fy axial scratching force, Fz normal scratching force. (**a**) 0 Hz, (**b**) 30 Hz, (**c**) 60 Hz, (**d**) 120 Hz, (**e**) average scratching force.

Scratching force in the scratching process of SiCp/Al reflects the removal mode and damage behavior of the material [37]. Therefore, examining the scratching force during the CS1 and NVAS processes is crucial. Figure 6 displays the variation of the scratching force during the CS1 and NVAS processes. The overall scratching force is low due to the shallow depth of the scratching, as embodied in Figure 6a–c. The variability of the scratching force in the three directions becomes larger with the enhancement of the vibration frequency compared with CS1, which may deteriorate the surface quality. As embodied in Figure 6d, under the vibration frequency of 120 Hz, the magnitude of changes in the scratching force of three directions decreases compared to CS1, and the scratching force fluctuates from −0.03 N to 0.1 N. As embodied in Figure 6e, scratch force data are processed in absolute values, and the mean value is calculated. The average normal scratching force increased from 0.045 N to 0.183 N compared to CS1. The average axial scratching force increased from 0.012 N to 0.18 N under the vibration frequency applied up to 30 Hz, which was due to the severe particle crushing, and the average normal scratching force decreased from 0.045 N to 0.033 N under the vibration frequency applied up to 120 Hz. The average axial scratching force decreases from 0.012 N to 0.004 N. This may be because the increase in vibration frequency lengthens the trajectory of the indenter, the time of interaction of the indenter with the sample is extended, the scratching force per unit of time decreases [38], and the increase in vibration frequency prevents the accumulation of debris in the scratching groove, which helps reduce the scratching force and friction [39].

In summary, there are fewer plasticity removal regions when the vibration frequency is lower compared to CS1. With a higher vibration frequency, the scratching process is dominated by plastic removal. Qualitative analyses of CS1 and NVAS were carried out using MD simulations to understand the effect of vibration frequency on the microplastic deformation mechanism.

### 3.2. MD Simulation of the Repercussion of Vibration Frequency on Scratching Results

The analysis of factors such as dislocations, stresses, and phase transitions is the key to a deeper understanding of the deformation mechanisms and damage behavior of materials under local external forces.

#### 3.2.1. Repercussion of Vibration Frequency on the Cross-Section of Scratched Grooves

**Figure 7:**
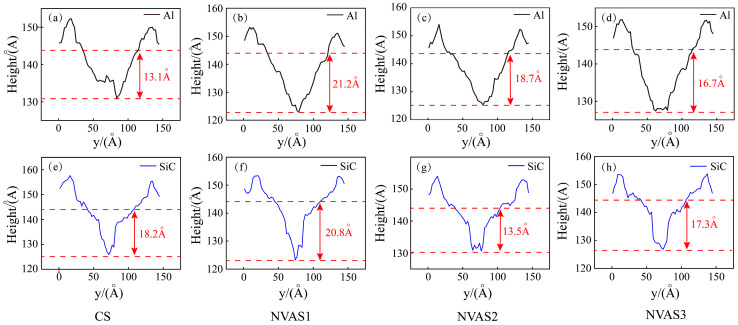
Cross-section of scratched grooves in SiC particles and Al matrix during CS and NVAS. (**a**–**d**) Scratching Al Matrix, (**e**–**h**) Scratching SiC particles.

As embodied in Figure 7, the cross-section was obtained by the OVITO post-processing function, and the two red dashed lines represent the positions of the bottom of the groove and the surface of the sample, which is at 144Å. The scratching groove cross-sections of SiC particles and Al matrix during CS and NVAS are shown, and the reason that the scratching depth is smaller than the set simulation depth may be the elastic recovery effect. As embodied in Figure 7a–d, the scratching depth of CS is 13.1 Å for scratching the Al matrix and 16.7 Å for scratching the Al matrix under the frequency of 100 GHz. The scratching depth of NAVS is much larger than that of CS. With rising frequency, the bottom of the scratching groove cross-section becomes increasingly smooth over time. As embodied in Figure 7e–h, the scratching depth of CS is 18.2 Å during the scratching of SiC particles, while the fluctuation degree of the scratching depth of NAVS is larger. The bottom of the scratching groove cross-section is concave and uneven for the given frequencies of 25 GHz and 50 GHz. For the given frequency of 100 GHz, the bottom of the scratched groove section is relatively smooth. When comparing the depth difference of the groove cross-section between the scratched Al matrix and SiC particles, it can be found that for the given frequency of 50 GHz, the depth difference is large, which makes it difficult to achieve uniform removal. For the given frequency of 25 GHz and 100 GHz, there is no significant difference in depth, with the depth difference of 0.4 Å and 0.6 Å, respectively, which suggests that for the given frequency of 100 GHz, the material can be removed uniformly by reducing the difference between the two phases and the depth difference between the two phases.

#### 3.2.2. Hydrostatic Stress, Dislocation, and Phase Transition Analysis

**Figure 8:**
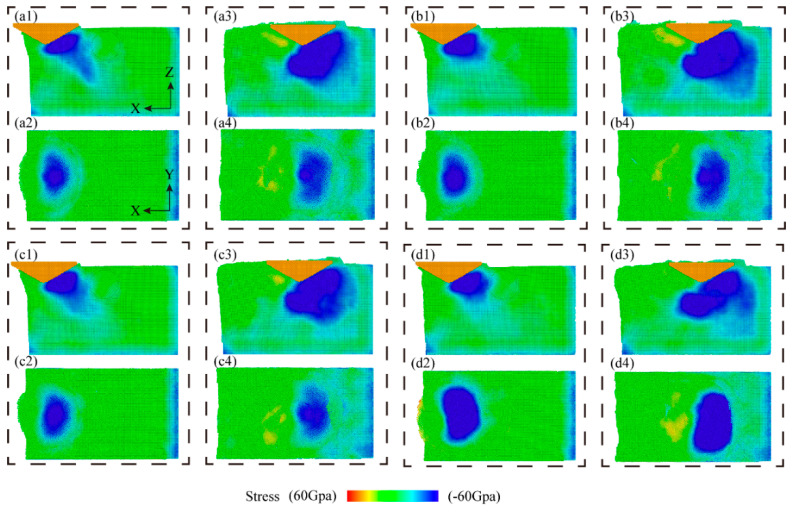
Distribution of hydrostatic stress during CS and NVAS processes: (**a1**–**d1**) display the hydrostatic stress distribution in the y-direction view while cutting the Al matrix, and (**a2**–**d2**) correspond to the same cutting process in the z-direction view. (**a3**–**d3**) show the hydrostatic stress distribution in the y-direction view during the cutting of SiC particles, and (**a4**–**d4**) correspond to the same cutting process in the z-direction view for SiC particles.

The stress dispersion prescribes the morphology of the material removal. Figure 8 shows the hydrostatic stress distribution during the scratching operation, where the hydrostatic stress in the scratched area is calculated by averaging the positive stresses applied to each atom in three directions. As embodied in Figure 8, the hydrostatic stress distributions under CS and NVAS are compared. Generally, the stresses during the scratching process are primarily focused on the forward and lower portions of the diamond indenter. As embodied in Figure 8(a1–d1), the hydrostatic stresses on the Al matrix are distributed more uniformly and fluctuate less as the vibration frequency increases. As embodied in Figure 8(a3–d3), when the indenter is applied to the SiC particles, the stresses rapidly extend to all SiC particles. As embodied in Figure 8(a2–d2,a4–d4), the hydrostatic stress distribution in the NVAS process is wider compared with that in the CS, which is probably because the vibration is applied in the axial direction and the material undergoes axial compression and shear in the NVAS process. Uneven stress distribution may lead to damage to the material. As the vibration frequency increases, the hydrostatic stress distribution in the NVAS process becomes more uniform. The enhanced range of hydrostatic stress distribution is beneficial in realizing the plastic deformation of the material [40].

**Figure 9:**
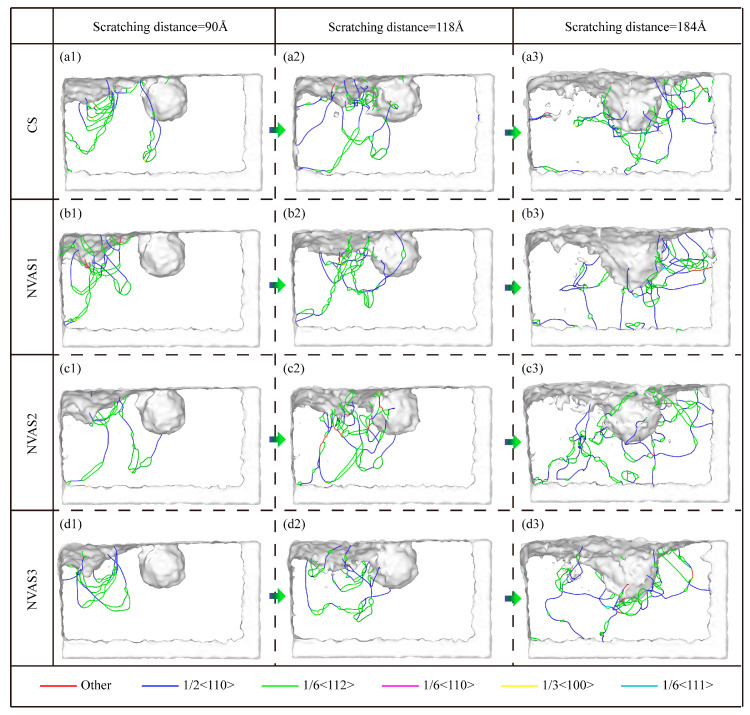
Dislocation analysis during CS and NVAS. (**a1**–**d3**) Distribution and evolution of dislocations in the Al matrix.

As embodied in Figure 9, the dislocations and phase transition analysis during CS and NVAS are shown. The dislocations are evenly dispersed throughout the Al matrix, which allows the slip system to be activated uniformly inside the crystal. The localized stress concentration and inhomogeneous deformation lead to the uneven distribution of dislocations in the matrix. As embodied in Figure 9(a1–d1), the dislocations are distributed inside the matrix at a scratching distance of 90 Å. In the CS process, the dislocations are focused within the touching area between the indenter and sample; the distribution of dislocations inside the matrix is more uniform with the increase in frequency. As a high number of dislocation loops will have a marked effect on the movement of dislocations [41], restricting the slip of dislocations, thus increasing the yield strength and decreasing the plastic deformation capacity of the material, a low number of dislocation loops implies that there exists less obstruction and the dislocations are free to slip. With increasing frequency, the amount of dislocation loops decreases significantly, which shows that the vibration improves the plastic deformation ability of the material. SiC particles hinder the movement of dislocations [42], and the dislocations are plugged between SiC particles and the Al matrix. As embodied in Figure 9(b2,c2), a large number of dislocation loops are generated when the frequencies are 25 GHz and 50 GHz, which shows that the lower frequency suppresses the plastic deformation of the material and when the frequency reaches the highest, the dislocation plugging is significantly improved. The dislocation distribution is more uniform. This is embodied in Figure 9(a3–d3), which shows the dislocation distribution around the SiC particles when the scratching distance is 184 Å. It can be seen that the dislocations are uniformly distributed around the SiC particles with the increase in the frequency.

**Figure 10:**
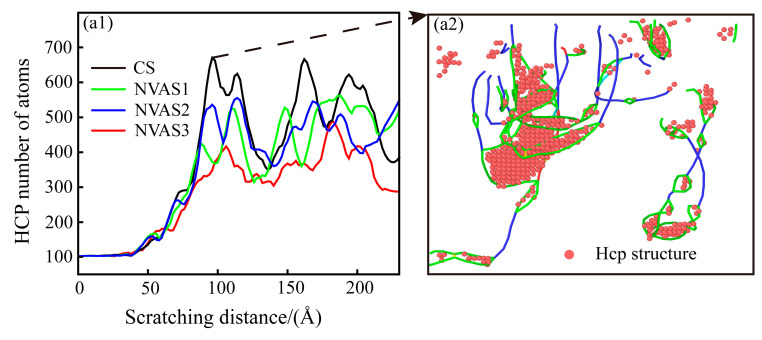
Phase transition analysis during CS and NVAS. (**a1**) Phase transition in Al matrix. (**a2**) HCP structure in dislocation loop.

It is shown that the FCC structure has more slip systems than the HCP structure [43], and the increased number of HCP structures usually represents the material’s restricted slip systems in plastic deformation, which results in the material exhibiting higher strength [44]. The phase transition of the scratching process is embodied in Figure 10(a1), which indicates that there is a phase transition from FCC to HCP in both the CS and NVAS processes, and the amount of HCP structures is significantly increased when the indenter contacts the Al matrix. The amount of HCP structures substantially increases when the indenter contacts the SiC particles. The number of HCP structures reduces with the increase in the vibrational frequency, which attests that the vibration is favorable for improving the plasticity of the material. As embodied in Figure 10(a2), the HCP structures mainly exist in the dislocation loop, which may be due to the plastic deformation of the Al matrix during the scratching process. Stress conditions and lattice warping in the vicinity of the dislocation loop are more considerable.

**Figure 11:**
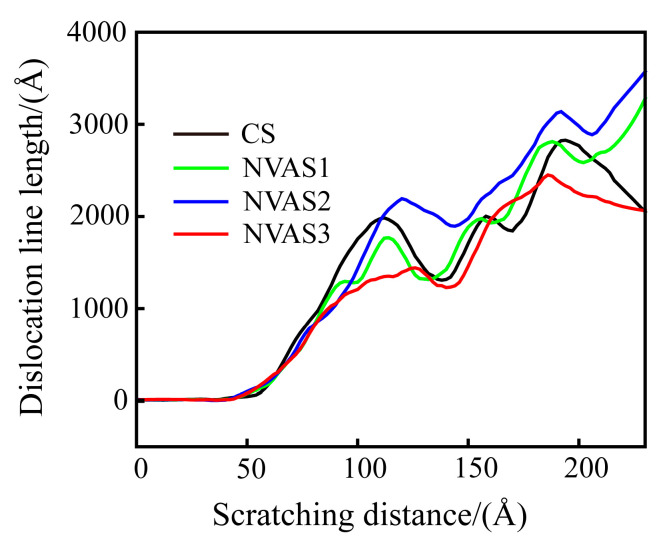
Variation of dislocation line length during CS and NVAS.

The dislocation density can quantitatively describe the magnitude of plastic deformation of the material during the scratching process [45]. Dislocation density is computed as the ratio between the length of the dislocation line and the volume of the model. Since the same volume of the model material is used in this paper, the length of the dislocation line is used to characterize dislocation density. As embodied in Figure 11, the dislocation density gradually increases during the scratching process, and the dislocation slip becomes difficult. Under the vibration frequencies of 25 GHz and 50 GHz, dislocation density is higher than that of CS, which manifests that the plastic deformation of the material is inhibited at a lower frequency. Under a frequency of 100 GHz, dislocation density falls below that of CS, which demonstrates that the dislocations reduce the obstruction of material deformation. The higher frequency promotes the plastic deformation of the material. Scratch experiments and MD simulations yielded consistent conclusions. As plastic deformation proceeds, the dislocation density increases, and the interaction between the dislocations prevents further movement of the dislocations, which leads to work hardening. A vibration frequency of 100 GHz improves the work-hardening phenomenon compared to CS. The work-hardening effect increases the material’s resistance to deformation, thus increasing the surface hardness.

#### 3.2.3. Effect of Vibration Frequency on Scratching Force

**Figure 12:**
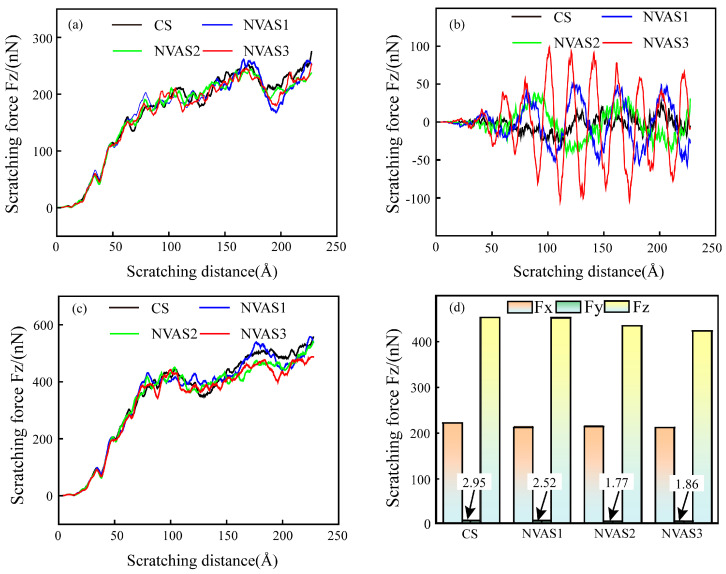
Variation of scratching force during CS and NVAS: (**a**) tangential scratching force, (**b**) axial scratching force, (**c**) normal scratching force, (**d**) average scratching force.

As embodied in Figure 10, the changes in scratching force during CS and NVAS are compared. As embodied in Figure 12a,c, tangential and normal forces increase with the rise in scratching distance, and the normal force is significantly greater than the tangential force. As embodied in Figure 12b, the maximum value of the axial force of NVAS is considerably larger than CS. The maximum value of the axial force becomes larger alongside the growth of the vibration frequency, which may be due to the introduction of vibration enhancement in the amount of contact between the indenter and atoms. The amount of contacting atoms is enhanced with the increased vibration frequency. The instantaneous axial scratching force can reach up to 97 nN under the vibration frequency of 100 GHz. The scratching force is periodic because the material is continually subjected to the continuous impact of the indenter [46], which is the reason for the wider hydrostatic stress distribution in the NVAS process. Scratching force fluctuates greatly during CS. As embodied in Figure 12d, to quantitatively analyze the scratching force in the CS and NVAS processes, the scratching force in the stable scratching stage is averaged. For the given vibration frequency of 100 GHz, the average tangential scratching force is 211 nN, the average axial scratching force is 1.86 nN, and the average normal scratching force is 424 nN. The instantaneous axial scratching force is larger; the average scratching force in all three directions decreases as the vibration frequency increases, which can realize better surface quality and is the same as the actual results of the scratching experiment.

## 4. Conclusions

To analyze the material removal mechanism of SiCp/Al under the action of non-resonant vibration, the effects of different vibration frequencies on the scratch morphology and scratch force of SiCp/Al were revealed by NVAS experiments, and the plastic deformation mechanism of SiCp/Al under different vibration frequencies was revealed by MD simulation.

(1) The increase in vibration frequency changes the material removal behavior. At 30 Hz, particle crushing intensifies and the plasticity removal area decreases. At 60 Hz, particle crushing worsens, but at 120 Hz, particle crushing is reduced, the plasticity removal area increases, and the scratching groove becomes smoother.
(2) Simulations show that frequencies of 12.5 GHz and 50 GHz significantly increase HCP structures and dislocation density compared to CS. A 100 GHz vibration frequency leads to more uniform SiC particle removal, improved smoothness of the scratching contour, wider hydrostatic stress distribution, more uniform dislocation distribution, and a reduction in dislocation loops and HCP structures. The phenomenon of dislocation plugging is improved considerably, and the density of dislocations is reduced, which contributes to plastic deformation.
(3) Higher vibration frequency reduces the scratching force. Compared to the CS, as the vibration frequency increases by 120 Hz, the average normal scratching force drops from 0.069 N to 0.0628 N, and the average axial scratching force decreases from 0.012 N to 0.006 N. According to the simulation results, the average axial scratching force is 1.86 nN, and the instantaneous axial scratching force can reach a maximum of 97 nN when the vibration frequency increases by 100 GHz.

In summary, this study provides a theoretical basis for low-damage machined surfaces of other difficult-to-machine particle-reinforced metal matrix composites.

## Figures and Tables

**Table 1:** SiCp/Al scratching experiment parameters.

Number	Scratching Velocity/(μm/s)	Scratching Depth/(μm)	Amplitude/(μm)	Frequency/(Hz)
CS1	100	0.2	-	-
CS2	100	3	-	-
CS3	100	6	-	-
NVAS1	100	0.2	5	30
NVAS2	100	0.2	5	60
NVAS3	100	0.2	5	120

**Table 2:** Detailed MD simulation parameters.

Properties	Parameters
Atomic number	318,743
Time step/(ps)	0.001
Scratching depth/(Å)	27
Scratching speed/(Å/ps)	2
Scratching distance/(Å)	250
Vibration amplitude/(Å)	3
Vibration frequency/(GHz)	0, 25, 50, 100

**Table 3:** Parameters of lj/cut potential.

System	Parameters (Unite)		
	ε (eV)	Σ (Å)	$r_{c}$ (Å)
$Si-C_{diamond}$	0.004553	3.851	10.0
$C_{sic}-C_{diamond}$	0.008452	4.103	10.0
$Al-C_{diamond}$	0.026342	3.48	10.0
Si−Al	0.026342	3.48	10.0
$C_{\mathrm{sic}}-Al$	0.038674	3.855	10.0

## Data Availability

The original contributions presented in this study are included in the article; further inquiries can be directed to the corresponding author.

## Abbreviations

The following abbreviations are used in this manuscript:

SiCp/Al	aluminum-based silicon carbide
CS	conventional scratching
NVAS	non-resonant vibration-assisted scratching
MD	molecular dynamics
PZT	piezoceramic
